# Effect of long-term cervical extensor exercise program on functional disability, pain intensity, range of motion, cervical muscle mass, and cervical curvature in young adult population with chronic non-specific neck pain: a randomized controlled trial

**DOI:** 10.1186/s13018-023-04487-w

**Published:** 2024-01-03

**Authors:** Yao Zhang, Wancheng Lin, Meng Yi, Jipeng Song, Lixiang Ding

**Affiliations:** Department of Spinal Surgery, Beijing Shijitan Hospital, Capital Medical University, No. 10, Tieyi Road, Yangfangdian, Haidian District, Beijing, 10038 People’s Republic of China

**Keywords:** Chronic non-specific neck pain, Cervical extensor exercise, Isometric training, Cervical lordosis, Cervical range of motion

## Abstract

**Background:**

The prevalence of chronic non-specific neck pain (CNNP) is on the rise among the young adult population. We herein aimed to compare the effects of long-term specific cervical extensor training and stretching exercises on improving this chronic disorder in young adults.

**Methods:**

In this prospective, randomized, controlled study, 70 participants aged 18–35 years with CNNP and cervical lordosis loss were included. The participants were assigned to undergo either specific cervical extensor training (observation group) or perform usual stretching exercises (control group). The exercise duration was set at 12 months, with 9 months at the clinic and 3 months at home. The outcome assessments included changes in the neck disability index, visual analog scale from baseline, cervical range of motion (CROM), cross-sectional areas (CSAs) of cervical extensors, and cervical curvature from baseline. The outcome measures were compared between groups at 3, 6, and 12 months of follow-up.

**Results:**

All 70 participants underwent randomization, and no significant differences in demographics and baseline data were found between the two groups. The observation group showed a greater improvement in neck disability index and visual analog scale scores at the 12-month follow-up than the control group. Additionally, a more substantial increase in CROM and CSAs of cervical extensors was observed in the observation group at the 6-month and 12-month follow-ups (*P* < 0.05). Although more participants in the observation group achieved cervical lordosis at the 12-month follow-up, the difference was marginally nonsignificant (9% in the control group vs. 28% in the observation group, *P* = 0.075).

**Conclusions:**

In young adults with CNNP, long-term specific cervical extensor training was associated with a more significant clinically meaningful improvement in disability, pain, and CROM than stretching exercises. The increased CSAs of cervical extensors may potentially contribute to the restoration of cervical lordosis.

*Trial registration* The study is registered at the Chinese domestic clinical trial (ChiCTR2000040009) at Chictr.org. The date of registration: November 18, 2020.

## Introduction

Non-specific neck pain stands as the fourth leading cause of chronic disability, with an annual prevalence rate exceeding 30% [1]. Chronic non-specific neck pain (CNNP), defined as pain persisting for 3 months or longer [2], is projected to affect 48%–67% of individuals at some point in their lifetime [3, 4]. The economic repercussions of neck pain extend to both individuals and society, encompassing costs related to healthcare, insurance, loss of productivity, and sick leave [5]. In young adults, neck pain has been identified as a risk factor for reduced general work productivity [6].

Young adulthood, a transitional stage between adolescence and adulthood or early adulthood and full social membership [7], introduces inequalities in socioeconomic status [8]. Simultaneously, biological parameters such as bone mass [9] and muscle strength [10] peak during young adulthood, rendering individuals vulnerable to future musculoskeletal health issues. Young adults are at a high risk of experiencing neck pain, with a 12-month prevalence ranging from 42 to 67% [11–13]. Hence, the phase of young adulthood assumes a pivotal role in shaping the long-term trajectory of musculoskeletal pain development and management. Ideally, interventions during this period would effectively diminish occurrences of neck pain and mitigate its consequential impact in adulthood [14].

The cervical extensors were organized into four layers [15], with the deepest layer, including semispinalis cervicis, multifidus, and rotatores, recognized as key stabilizers for cervical spine segmental support [16]. Emerging evidence suggests variable changes in cervical extensor muscles among individuals with CNNP [17]: remodeling of musculature structures, such as larger cross-sectional areas (CSAs) of superficial layer extensors and smaller CSAs of deep layer extensors [18, 19], transformation from type I to type II fibers [20], and alterations in muscle behavior (increased and decreased activation in superficial and deep cervical extensors, respectively) [21, 22]. Furthermore, deficits in cervical muscle activity may lead to poor joint movement control, repeated microtrauma, and subsequent pain, resulting in decreased cervical range of motion (CROM) and cervical lordosis loss [17, 23].

Conservative care for patients with neck pain often includes pharmacologic therapies. While practice patterns may favor the use of specific agents, such as nonsteroidal anti-inflammatory drugs, corticosteroids, and opioid analgesics, providing short-term pain relief, there is limited evidence supporting their long-term use in most patients with CNNP [24–26]. Exercise is a crucial component of treatment programs for patients with CNNP [27, 28]. Several trials have concluded that 1- to 6-month neck stretching exercises can decrease neck pain and improve neck function in 20–50-year-old office workers suffering from chronic moderate-to-severe neck pain [29–31]. However, stretching exercises may be insufficient in improving muscle strength [31]. A few trials have studied the effectiveness of specific or non-specific cervical extensor exercises in alleviating neck pain and disability, increasing CSAs of extensors, enhancing neck muscle strength and functions, and improving cervical curvature and CROM [32–35].

To the best of our knowledge, no previous study has investigated the comprehensive treatment effect of a long-term specific training program for cervical extensors in young adults with CNNP. We herein aimed to test the hypotheses that the 12-month exercise program for cervical extensors combined with one-week drug therapy in young adults with CNNP relieves neck pain, improves neck disability, increases CROM and CSA of extensors, as well as restores cervical lordosis.

## Methods

### Study design and oversight

This single-blind, single-center, prospective, randomized controlled trial was conducted at Beijing Shijitan Hospital and approved by the Institutional Ethics Committee of Beijing Shijitan Hospital (2020-81-K47). The trial adhered to the requirements of the Declaration of Helsinki and was registered as a Chinese domestic clinical trial (ChiCTR2000040009) at Chictr.org. All enrolled participants provided written informed consent. Participants were recruited from the spinal surgery clinics of our institution by the two corresponding authors between December 1, 2020, and August 1, 2021. Costs during the trial, including imageological examinations, medications, and therapeutic training, were covered by the research team and financed by the National Key R&D Plan (2022YFC3600402). The trial was limited to 1-year follow-up data due to the constraints related to research fellows, complexity of research progress, and high dropout rates. Data management was handled by two investigators (WC. L and M. Y) not involved in the randomization and intervention process. All authors vouched for the completeness and accuracy of the data and the trial’s fidelity to the protocol.

### Participants

Individuals aged 18–35 years who visited the spinal surgery clinic for CNNP of more than 3 months were eligible for enrollment. The following participants were included: 1. Those with a neck disability index (NDI) of ≥ 50% and a visual analog scale (VAS) of ≥ 4 (indicating at least moderate pain and disability) and 2. Those identified with straight or kyphotic cervical curvature (cervical curvature defined as “kyphotic” for an angle of >  + 4°, “straight” for an angle between − 4° to + 4°, or “lordotic” for an angle of <  − 4° from the lateral view of radiography using the posterior tangent technique, as described by Albers [36] and Gore [37]). The following participants were excluded: 1. Having a history of cervical spine surgery or trauma (e.g., whiplash injury, fracture, and dislocation); 2. Showing signs of medical “red flags” (infection, tumors, rheumatic arthritis, or cardiovascular disease); 3. Diagnosed with cervical radiculopathy or myelopathy (the myotomal strength, sensation, or reflexes were confirmed to be diminished upon physical check-up, which in conformity to the involvement of responsible segment of nerve root or spinal cord on imageological results); 4. Currently taking pain medications, undergoing physiotherapy treatment, or participating in neck or shoulder exercise programs within the last 6 months; 5. Allergic to opioid or non-steroidal anti-inflammatory drugs; 6. Suffering from severe acute neck pain or having psychological disorders.

Carefully medical history taking and physical check-up are of great importance for identifying the eligible participants. Moreover, the X-ray and magnetic resonance image of cervical spine were obtained at this stage, participants with non-specific neck pain can be accurately confirmed. For ensuring the trial’s quality, the two most experienced researchers (LX. D: physiotherapist with more than 10 years of clinical experience, and JP. S: physiotherapist with 5 years of clinical experience) were assigned to oversee the inclusion and exclusion of participants.

### Randomization and blinding

Participants were randomly assigned to the control group or observation group in a 1:1 ratio in the clinic by the two corresponding authors (LX. D and JP. S). During randomization, random numbers were selected from a random number table and concealed in opaque envelopes. Participants with odd numbers were assigned to the control group and those with even numbers were assigned to the observation group. The participants, data collectors, and statistical analysts were blinded to the treatment, but the trainers were aware of the intervention due to the trial’s design. To minimize exposure between groups, participants from different groups underwent intervention on different days.

### Intervention

All participants were given compound codeine phosphate and ibuprofen sustained release tablets (0.2 g each tablet, 2 tablets each time, once a day) for 1 week to ameliorate acute pain, conducive to further exercise intervention. Simultaneously, all participants performed specific cervical extensors exercises or usual cervical extensors stretching exercises thrice a week for 12 months. Participants in both groups were encouraged to exert maximal effort during all exercises. The exercises implemented in both groups were derived from previous research [17, 31, 32, 38]. Due to the designated long duration of the exercise program, the training program from previous trials was modified and simplified to some extent.

In the first 9 months, the participants were required to visit our clinic and perform exercises under instruction and supervision. All exercise processes were guided by three researchers (LX. D, JP. S, and Y. Z) with more than 5 years of experience in musculoskeletal disorder treatment. In the later 3 months, participants executed the same exercise program at home, with follow-up and supervision provided by two researchers (JP. S and Y. Z) weekly through video calls.

#### Control group

The participants in this group underwent the usual neck muscle stretching exercise program, which was a modified version of the training protocol from previous research [29, 31] and designed to be easily learned and performed at home. The 12-month exercise in the control group were instructed and supervised by JP. S and Y. Z.

**Fig. 1:**
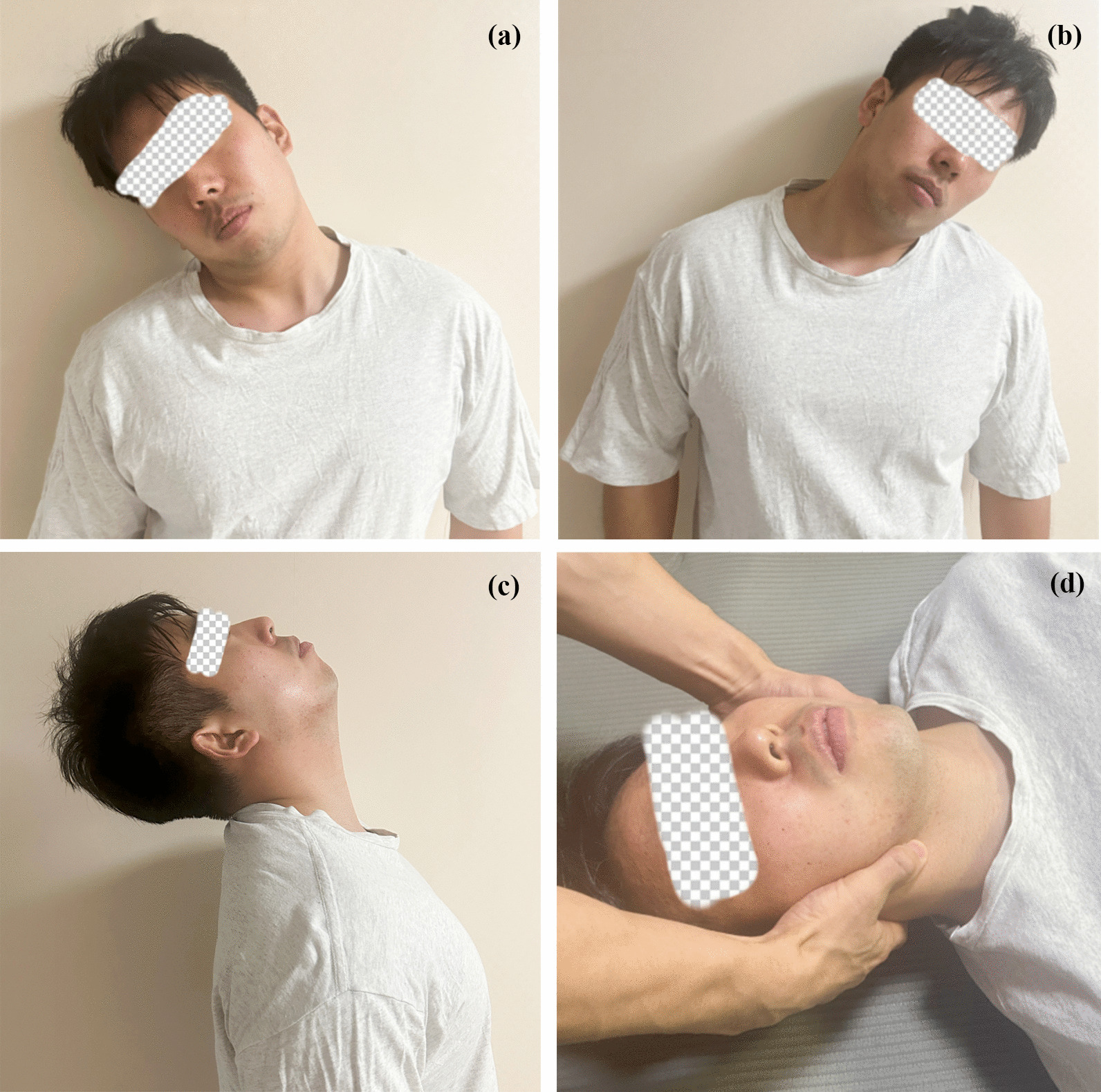
Neck stretching exercise for the control group; **a**, **b**, **c**: three-direction active neck stretching exercise; **d**: manual traction of the cervical spine

Initially, the participant was asked to sit on a stool and rotate the head freely for 5 min to relax and warm up the cervical muscles. Subsequently, the participant repeatedly bent the neck 15 times per set, four sets per direction with a 30-s rest between sets, for three directions. Finally, the participant lay supine to undergo manual traction of the cervical spine for 10 min (Fig. 1).

#### Observation group

The participants in the observation group underwent specific cervical extensor training, including isometric and isokinetic exercises, referencing the study of Schomacher et al. [39]. Additionally, for the convenience of at-home exercise, the training protocol was a modified and simplified version of previous exercise programs from Tsang et al. [32] and Giménez-Costa et al. [33]. The warm-up procedure was the same as that in the control group. The first 9-month exercise (in clinic) were instructed and supervised by LX. D and JP. S; JP. S and Y. Z were responsible for the later 3-month exercise (at home).

**Fig. 2:**
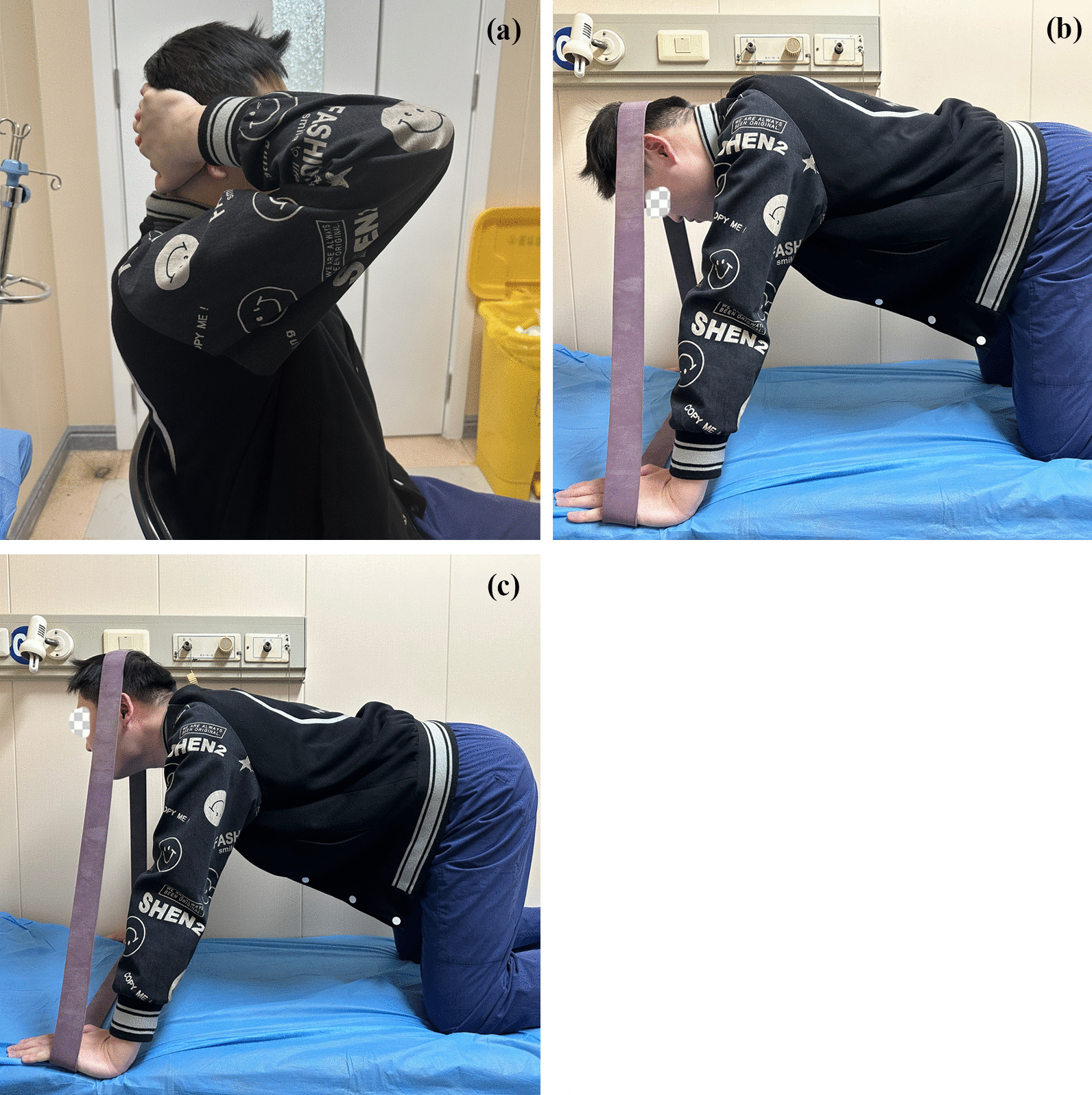
Cervical extensors exercise in the observation group; **a** isometric extensor training; **b**, **c** isokinetic extensor training

For isometric training focusing on deep cervical extensors, the participant sat upright on a chair, hands folded and placed slightly below the occiput. The participants then pushed into extension against the resistance applied to their hands and were encouraged to maintain this posture as long as possible, with a 30-s rest allowed when feeling fatigued. The participants engaged in extensor isometric training for 15 min. After a 5-min rest, they performed isokinetic exercise focusing on general cervical extensors. During the exercise, the participant knelt on the bed with arms straightened and hands on the bed to maintain steadiness (4-point kneeling position). A resistance band was placed under the hands and surrounded the head (the location of the resistance applied at the occiput). The participants could adjust suitable resistance by widening or shortening the distance between two hands. After all these preparations, the participants raised and lowered the head 15 times/set with an even speed, maintaining the tension of the resistance band, with a 30-s rest between sets for four sets. After the training, the participants underwent manual traction the same with the control group (Fig. 2).

### Outcome measures

All observation items (including clinical and imageological measures) were evaluated at the baseline and 3, 6, and 12 months after the trial commencement. At each follow-up, the participants were recruited to the clinic for outcomes measurement. Radiography and magnetic resonance images from each participant were reviewed by two researchers (LX. D and JP. S) to verify the classification of cervical curvature, as well as to measure the CROM and CSA of cervical extensors.

#### Functional disability

The perceived level of functional disability due to participant’ neck pain was assessed with the NDI, which was specified as the primary outcome measure. The NDI is a 10-element self-assessment instrument for evaluating the specific functional status of subjects with neck pain, encompassing pain, personal care, weight gain, reading, headache, concentration, work, driving, sleeping, and leisure. Each section is rated on a scale of 0–5, where 0 means “painless” and 5 means “the worst pain imaginable.” The points obtained are summed to a total score, interpreted as a percentage. The disability categories for NDI are 0–8%, without disability; 10–28%, mild; 30–48%, moderate; 50–64%, serious; and 70%–100%, complete. The minimal clinically important difference, prespecified at 6.4, was determined based on previous research [40, 41].

#### Pain intensity

The level of pain intensity was measured using the disease-specific VAS score (range, 0–10, with higher scores indicating more neck pain). The minimal clinically important difference for the VAS score was 2.76 points, as determined by previous studies [42].

#### Cervical curvature

As previously described, cervical curvature was defined as “kyphotic” (angle >  + 4°), “straight” (angle between − 4° to + 4°), or “lordotic” (angle <  − 4°) from the lateral view on cervical radiography using the posterior tangent technique by Albers [36] and Gore [37].

#### CROM

**Fig. 3:**
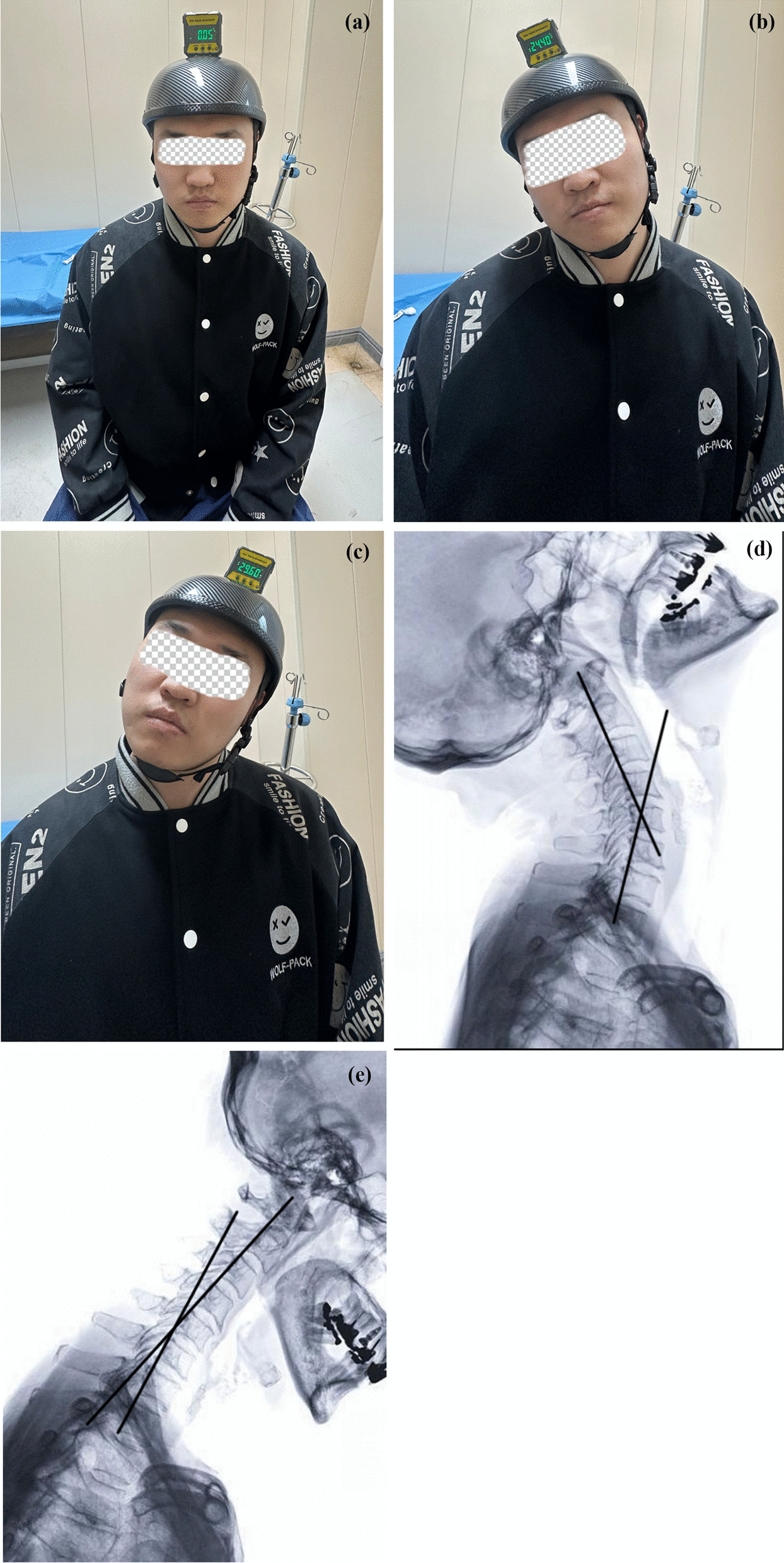
Two measurement methods of the CROM

The measurement of CROM was conducted by LX. D, JP. S, and Y. Z. To reduce measurement duration and improve accuracy, two methods were employed for measuring CROM. First, the CROM for extension and flexion was radiographically determined by calculating the difference value of cervical curvature between extension and flexion radiography. Next, the CROM of rotation and side-bending was measured by a specific device consisting of a lightweight helmet and a digital inclinometer. During the measurement, the participant sat on a chair with both upper and lower back in contact with the bracket and received clear instructions regarding the required movements, which included side bending (left and right) and rotation (left and right). The participant executed the movement on one side and maintained a stationary position for recording the values on the inclinometer. The values on the opposite side were recorded similarly. The summation of the absolute values on each side was considered as the CROM for rotation or side-bending (Fig. 3). Measurements were conducted three times for both rotation and side-bending movements, and the values were averaged and adopted.

#### CSAs of cervical extensors

**Fig. 4:**
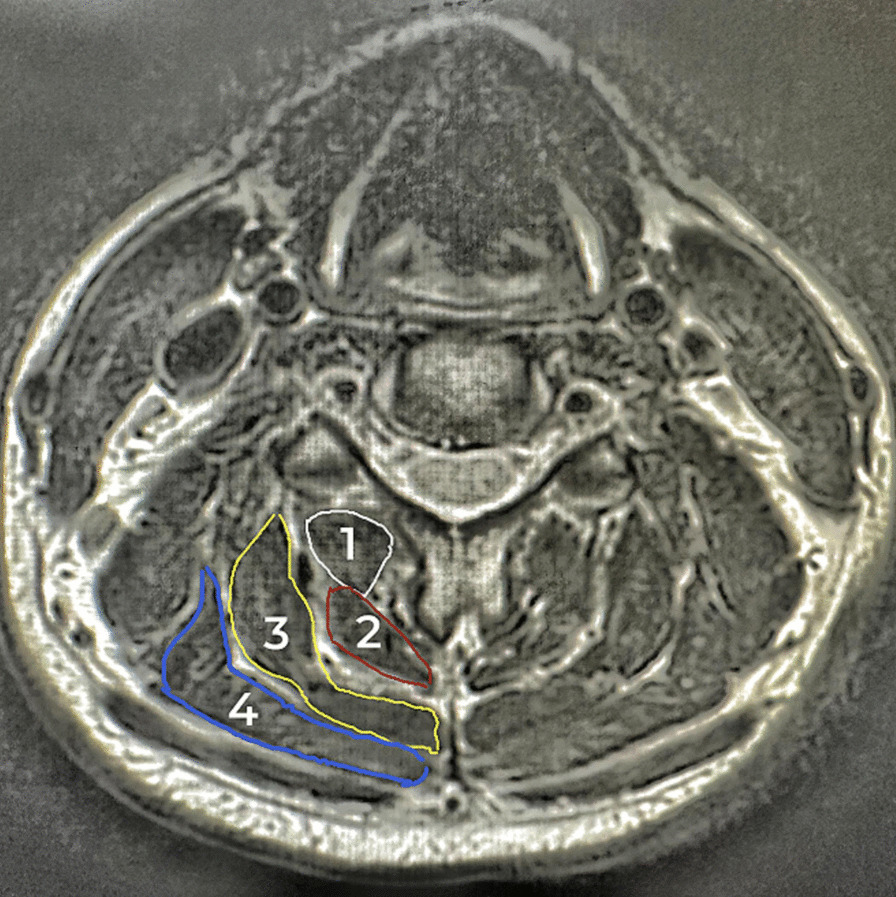
Measurement of total CSAs for the cervical extensors. 1: multifidus, 2: semispinalis cervicis, 3: semispinalis capitis, and 4: splenius capitis

Quantitative measurements of the CSAs of cervical extensors (including cervical multifidus, semispinalis cervicis, semispinalis capitis, and splenius capitis muscles) were taken from T1-weighted axial magnetic resonance images using the Picture Archiving and Communication System of our medical institution. The CSAs of cervical extensors were bilaterally measured at the mid-disc level of C2-C3, C3-C4, C4-C5, C5-C6, and C6-C7; the sagittal view was used for locating the corresponding mid-disc segment. Moreover, the multifidus and semispinalis cervicis were collectively measured at C2-C3 due to the substantial peri-articular fat and the absence of distinguishable muscle boundaries at this segment. The mean value of the sum of the CSAs on each side at all levels was calculated three times, and the resulting values were averaged and adopted (Fig. 4).

### Statistical analysis

The sample size estimation was based on previous nonrandom prospective or retrospective studies [38, 43] and was calculated using the SPSS 2021. We assumed a mean standard deviation of 3.5 for the change in NDI and a 10% rate of loss to follow-up at 1 year. With a sample size of 66 participants (33 participants in each group), the trial would have 90% power to detect an inter-group difference of 6.4% improvement in NDI score at a two-sided significance level of 0.05.

The primary analysis encompassed all participants who could be assessed in the per-protocol population (i.e., participants who had completed at least the 3-month follow-up). We utilized mixed-effects models for repeated measures of between-group comparisons of changes in the NDI scores, VAS scores, CROM measurements, CSAs measurements, and cervical curvature measurements from baseline. Fixed effects were included for the intervention manner, time (3 months, 6 months, and 12 months after the trial commencement), and the time × treatment interaction. Least-squares means and 95% confidence intervals (CI) were calculated, and robust standard errors and test statistics involving the fixed effects were computed using the PROC MIXED procedure (SAS Institute). Least-squares means between groups at each follow-up time point and within each group between follow-up time points were compared using appropriate contrasts within the mixed-effects models for repeated measures. For other analyses, independent-sample t-tests were performed for continuous variables, presented as means and SDs, and the χ^2^-test or Fisher’s exact test was performed for categorical variables, presented as numbers and percentages.

## Results

**Fig. 5:**
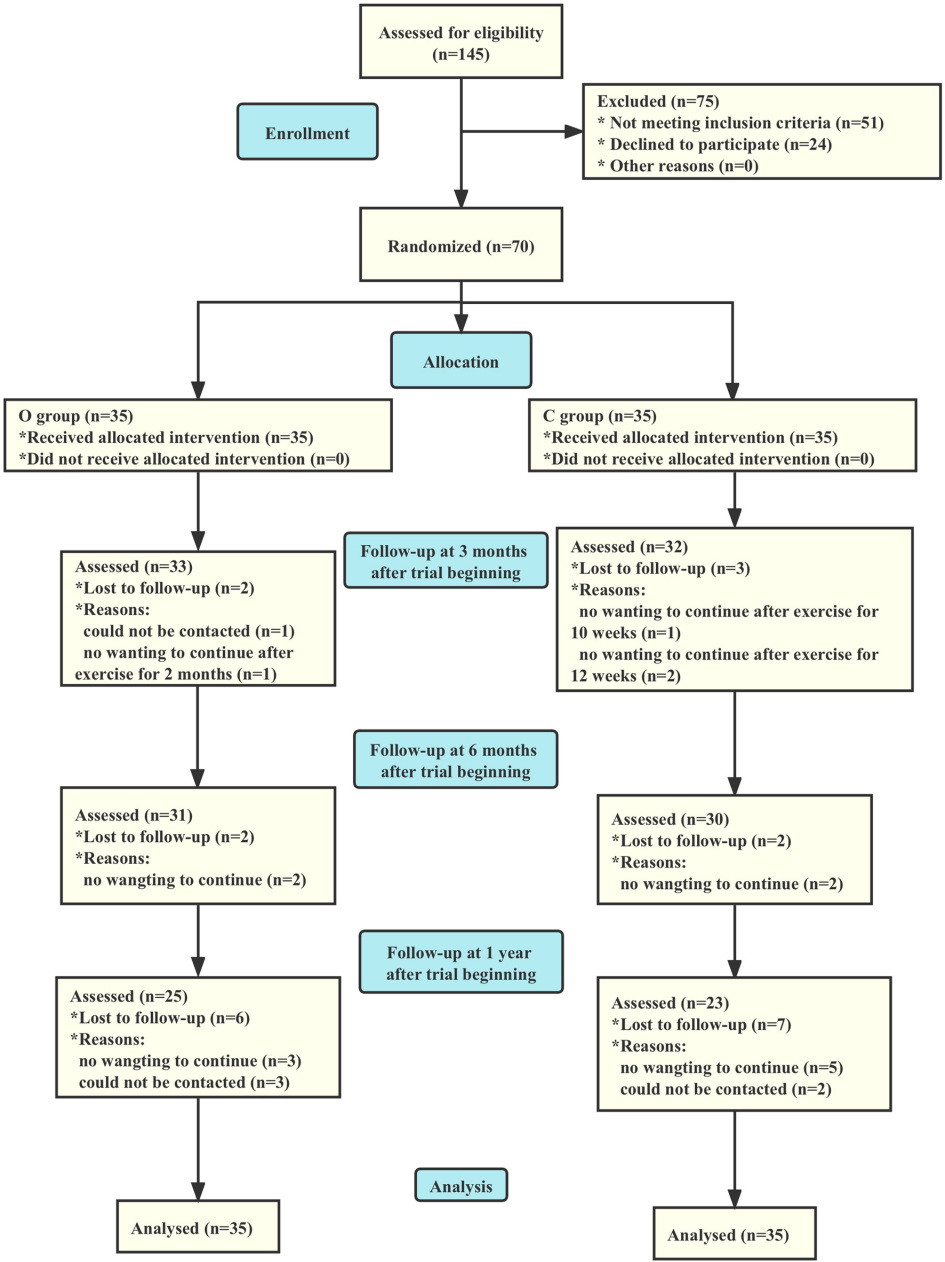
Eligibility, randomization, and follow-up; O group indicates observation group, C group indicates control group

The enrollment, randomization, and follow-up for this trial are depicted in Fig. 5. A total of 145 subjects were screened and identified as eligible, with 51 not meeting the inclusion criteria and 24 refusing to join. Consequently, 70 subjects (44 females and 26 males, mean age: 29 years) consented to randomization, with 65 completing at least a 3-month follow-up and being included in this analysis. Of the 65 analyzed participants, 61 completed a 6-month follow-up, and 48 completed a 12-month follow-up. The baseline characteristics were comparable between the two groups, as shown in Table 1. The dropout rate at the 3-month, 6-month, and 12-month follow-up was 7.1%, 12.9%, and 31%, respectively. Four participants in the observation group and two in the control group could not be contacted without reasons, and six participants in the observation group and ten in the control group declined to continue the exercise due to time and energy consumption related to long-distance trips. No adverse effects regarding drugs and exercise were observed at each follow-up.

**Table 1:** Demographics and baseline data of participants from two groups

Variables	Control group (*n* = 35)	Observation group (*n* = 35)	*P* value
Age (years)	29.46 ± 8.33	28.95 ± 7.86	0.703
Gender ratio (female/male)	1.50 (21/14)	1.92 (23/12)	0.647
BMI	26.18 ± 6.08	27.43 ± 5.78	0.744
Occupation proportion (quantities)			
Student	25.7% (9)	31.4% (11)	0.167
Medical stuff	17.1% (6)	14.3% (5)	0.426
Manual worker	14.3% (5)	20.0% (7)	0.601
Office clerk	28.6% (10)	22.9% (8)	0.454
Teacher	14.3% (5)	11.4% (4)	0.362
Duration of neck pain (weeks)	20.15 ± 5.26	19.66 ± 6.15	0.807
Cervical curvature			
Kyphotic	8 (22.9%)	10 (28.6%)	0.391
Straight	27 (77.1%)	25 (71.4%)	0.552
VAS	5.54 ± 1.01	5.31 ± 1.26	0.363
NDI (%)	35.45 ± 3.35	34.68 ± 3.03	0.206
CROM			
Rotation	53.16 ± 10.03	55.08 ± 9.60	0.195
Side-bending	65.08 ± 15.22	63.82 ± 13.48	0.219
Extension-flexion (degrees)	13.62 ± 4.77	15.12 ± 5.48	0.109
CSAs of cervical extensors (mm^2^)			
Summation of extensors	662.63 ± 178.23	635.06 ± 181.56	0.110

BMI: body mass index; VAS: visual analog scale; NDI: neck disability index; CROM: cervical range of motion; CSAs: cross-sectional areas

Partial data loss is allowed in the mixed-effects models; thus, participants who had completed at least a 3-month follow-up (65 participants, 32 in the control group and 33 in the observation group) were included in the outcome analysis. According to the prespecified value for the minimal clinically important difference (6.4), the NDI scores in the control group significantly improved at the 6-month follow-up (8.23) and continued to improve at the 12-month follow-up (12.69). In contrast, the observation group showed significant improvement at the 3-month follow-up (6.56), continuing at the 6-month follow-up (12.04), as well as at the 12-month follow-up (19.25). Additionally, a significant difference in the least squares means of the change in the NDI score between the groups was found at the 12-month follow-up (− 12.69 vs. − 19.25 points, difference: − 6.56, 95% CI − 17.63 to 4.51, *P* = 0.022) (Table 2).

**Table 2:** Changes in the clinical outcome measures including NDI, VAS scores, cervical ROM, and CSAs from the baseline

Outcomes	Change from baseline		Difference in change, control vs. observation (95% CI)	*P* value
	Control group (*n* = 32)	Observation group (*n* = 33)		
NDI (%)				
Baseline^a^	35.45	34.68	NA	NA
3-month follow-up	− 5.33	− 6.56	− 1.23 (− 8.13 to 5.67)	0.187
6-month follow-up	− 8.32	− 12.04	− 3.72 (− 12.13 to 4.69)	0.166
12-month follow-up	− 12.69	− 19.25	− 6.56 (− 11.97 to − 1.15)	0.022^*^
VAS				
Baseline^a^	5.54	5.31	NA	NA
3-month follow-up	− 1.28	− 1.21	0.07 (− 0.68 to 0.82)	0.281
6-month follow-up	− 2.13	− 2.45	− 0.32 − 1.23 to 0.59)	0.098
12-month follow-up	− 2.89	− 3.32	− 0.43 (− 0.82 to − 0.04)	0.035^*^
ROM				
Rotation				
Baseline^a^	53.16	55.08	NA	NA
3-month follow-up	5.43	6.17	0.74 (− 1.13 to 2.61)	0.165
6-month follow-up	18.51	27.29	8.78 (2.05 to 15.51)	0.021^*^
12-month follow-up	29.00	37.41	8.41 (1.74 to 15.08)	0.039^*^
Side-bending				
Baseline^a^	65.08	63.82	NA	NA
3-month follow-up	5.09	7.61	2.52 (− 1.33 to 6.37)	0.166
6-month follow-up	11.39	18.82	7.43 (1.26 to 13.60)	0.031^*^
12-month follow-up	18.72	26.20	7.48 (1.56 to 13.40)	0.029^*^
Extension-flexion (degrees)				
Baseline^a^	13.62	15.12	NA	
3-month follow-up	2.65	5.21	2.56 (− 0.85 to 5.97)	0.102
6-month follow-up	4.03	7.56	3.53 (0.86 to 6.20)	0.047^*^
12-month follow-up	4.96	9.71	4.75 (1.41 to 8.09)	0.038^*^
Total CSA of extensors (mm^2^)				
Baseline^a^	622.63	635.06	NA	NA
3-month follow-up	3.26	51.53	48.27 (16.73 to 79.81)	0.033^*^
6-month follow-up	4.23	76.36	72.31 (11.21 to 133.04)	0.021^*^
12-month follow-up	4.57	97.76	93.19 (17.29 to 169.09)	0.001^*^

Data are presented as least-squares mean values of changes in NDI, VAS scores, cervical ROM and CSAs from baseline at each follow-up time point. a**:** The baseline scores shown are the mean values in the group. NA indicates not applicable. *statistical significant

The participants in both groups demonstrated improvement in neck pain at each follow-up time point, assessed by VAS measurement. However, according to the prespecified minimal clinically important difference on a VAS score of 2.76 points, a significant difference for the two groups was only found at the 12-month follow-up from baseline (2.89 in the control group and 3.32 in the observation group). At the 6-month follow-up, there were marginal, non-significant differences in the least squares means of the change in the VAS score between the two groups, with *P* values between 0.05 and 0.1 (*P* = 0.098). A significant difference in the least squares means of the change in the VAS score between the groups was found at the 12-month follow-up (− 2.89 vs. − 3.32 points, difference: − 0.43, 95% CI − 1.92 to 1.06, *P* = 0.035) (Table 2).

Regarding the measurement of CROM (including rotation, side-bending, and extension-flexion), participants in both groups showed improvement at each follow-up time point. For the rotation component, a significant difference in the least squares means of the change between the groups was found at the 6-month follow-up (18.51 vs. 27.29, difference: 8.78, 95% CI 2.05–15.51, *P* = 0.021) and at the 12-month follow-up (29.00 vs. 37.41, difference: 8.41, 95% CI 1.74–15.08, *P* = 0.039). Similarly, for the side-bending and extension-flexion components, a significant difference in the least squares means of the change between the groups was found at the 6-month follow-up (11.39 vs. 18.82, difference: 7.43, 95% CI 1.26–13.60, *P* = 0.031, and 4.03 vs. 7.56, difference: 3.53, 95% CI 0.86–6.20, *P* = 0.047, respectively) and at the 12-month follow-up (4.96 vs. 9.71, difference: 4.75, 95% CI 1.41–8.09, *P* = 0.039, and 18.72 vs. 26.20, difference: 7.48, 95% CI 1.56–13.40, *P* = 0.029, respectively) (Table 2).

Extensive fatty infiltration of muscle tissue was not observed on magnetic resonance imaging before exercise and at the 12-month follow-up. The participants in the control group showed a slight increase in muscle areas at each follow-up time point from baseline, particularly from the 6-month follow-up to the 12-month follow-up. In contrast, the participants in the observation group had a greater increase in the CSAs of cervical extensors at every follow-up from baseline. A significant difference in the least squares means of the change between the groups was observed at each follow-up (Table 2).

**Fig. 6:**
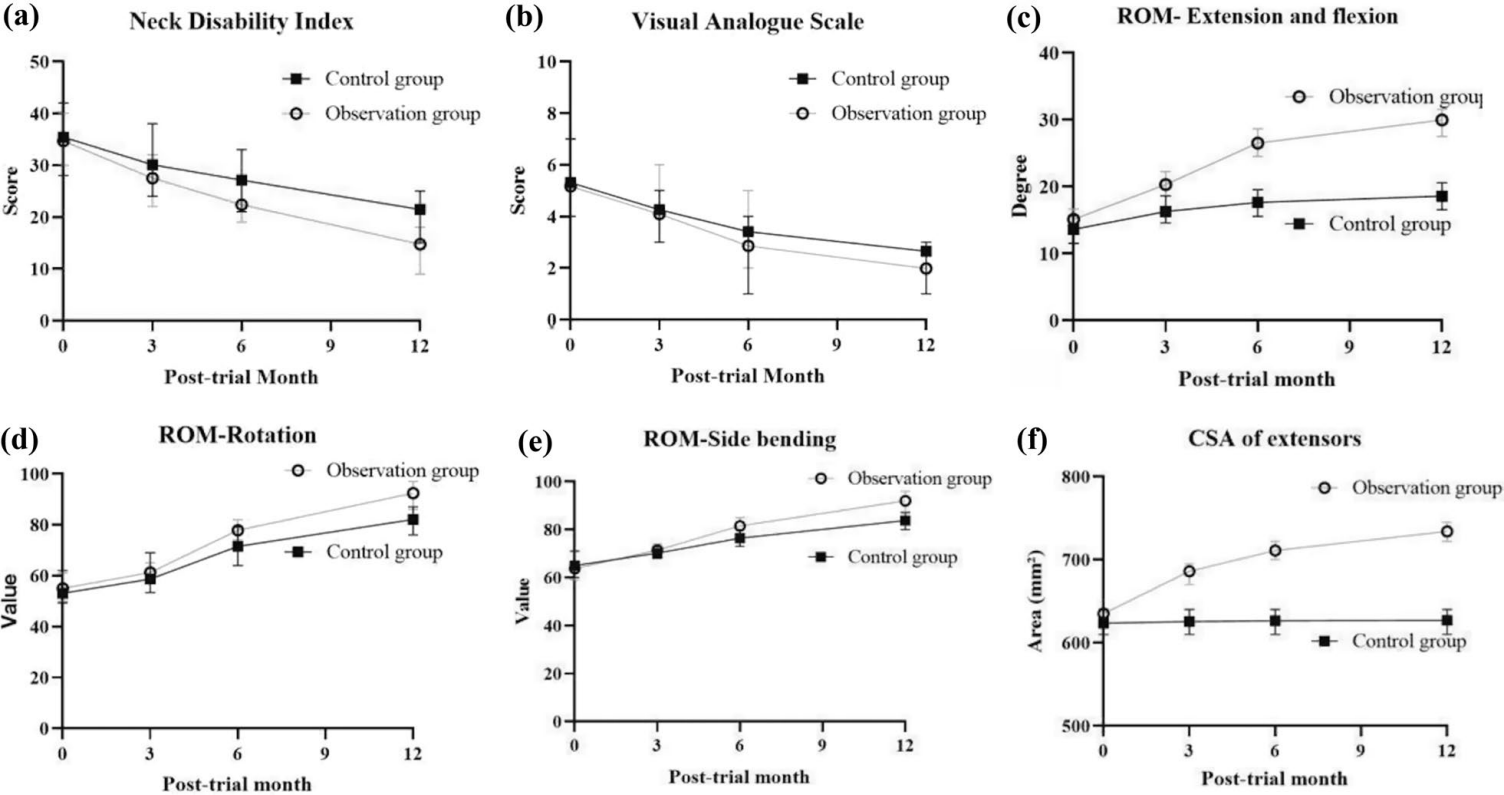
Variation tendency of NDI (panel **a**), VAS (panel **b**), CROM (panel **c, d, e**), and CSAs of cervical extensors (panel **f**) for two groups. I bars represent standard errors

The changes in cervical curvature from baseline for the two groups are shown in Table 3. No significant differences were observed in the proportion of participants with cervical lordosis between the two groups at each follow-up (*P* > 0.05). However, a marginal, nonsignificant difference between the two groups was found at the last follow-up (0.28 for the observation group vs. 0.09 for the control group, *P* = 0.075). The variation tendency of each measurement item is illustrated in Fig. 6.

**Table 3:** Cervical curvatures in the two study groups at different follow-up time points

	Control group			Observation group			*P* value^a^
	Kyphotic	Straight	Lordotic	Kyphotic	Straight	Lordotic	
Baseline^b^	8 (0.23)	27 (0.77)	0 (0.00)	9 (0.29)	26 (0.71)	0 (0.00)	0.782
3-month^c^	7 (0.22)	25 (0.78)	0 (0.00)	7 (0.21)	25 (0.76)	1 (0.03)	0.774
6-month^d^	5 (0.16)	25 (0.81)	1 (0.03)	6 (0.19)	22 (0.71)	3 (0.10)	0.836
12-month^e^	4 (0.17)	17 (0.74)	2 (0.09)	2 (0.80)	16 (0.64)	7 (0.28)	0.075

NA: not applicable; a: comparison of the proportion of participants with lordotic curvature between groups at different follow-up time points; b: 70 participants (35 in the control group, 35 in the observation group) were included in the statistical analysis at the baseline; c: 65 participants (32 in the control group, 33 in the observation group) were included in the statistical analysis at the 3-month follow-up; d: 61 participants (30 in the control group, 31 in the observation group) were included in the statistical analysis at the 6-month follow-up; e: 48 participants (23 in the control group, 25 in the observation group) were included in the statistical analysis at the 12-month follow-up

## Discussion

People in young adulthood is at a risk of developing neck pain [3, 44], a condition associated with significant suffering and financial costs [45]. Young adulthood emerges as a critical period during which the long-term development and management of musculoskeletal pain can be influenced. An easily implementable and effective exercise program would ideally reduce occurrences of neck pain and its consequences in adulthood. Several trials have demonstrated the efficacy of specific cervical extensor exercises in pain relief, disability improvement, muscle strength enhancement, and cervical lordosis restoration in patients with CNNP [32, 33, 35, 38, 46]. Our 12-month follow-up results have validated our hypothesis that, in young adult population with NCCP, a long-term specific training of cervical extensors is superior to usual stretching exercises in improving disability, pain, and CROM and increasing muscle CSAs.

Neck stretching exercises are a commonly utilized pain-relieving therapy for patients with CNNP. In this trial, participants from the control group underwent stretching exercises for 12 months (9 months at the clinic and 3 months at home), and continuous improvement in neck disability and relief of neck pain were observed during the 12-month follow-up. The findings in our control group differed from those of Häkkinen et al. [31]. In their study, the participants did stretching exercises at home, with a frequency of 5 times a week for 4 weeks. They observed that pain decreased significantly during the first 4 weeks but stabilized thereafter. In the study of Shariat et al. [29], the participants received stretching exercises under instruction, and pain relief was observed to be continuous during the 6-month follow-up. These findings highlight the influence of the training protocol on the efficacy of exercise. In this study, the participants in the observation group performed specific cervical extensor exercises, with modified isometric and isokinetic exercises, referencing previous exercise protocols from Tsang et al. [32] and Giménez-Costa et al. [33]. Our findings support the notion that specific extensor exercises are superior to stretching exercises in long-term pain and disability improvement, as significant between-group differences in NDI and VAS scores were only found at the 12-month follow-up. The duration of extensor exercise in this trial was the longest compared with similar studies [32, 33, 35, 38]. Additionally, our findings also confirmed that this modified training protocol is safe, as no adverse effects regarding exercise or drugs were observed during the 12-month follow-up.

In the present study, we measured the CROM on both radiography and a specially-made apparatus, instead of using the commonly used CROM instrument (Performance Attainment Associates, Roseville, USA) [46, 47]. Our measurement method has several advantages over the traditional one. First, we assessed the CROM of extension-flexion using lateral radiography, which is more objective and convenient; additionally, this will streamline the subsequent procedure for measuring rotation and side-bending. Second, the apparatus utilized in this study is a carbon-fiber, self-adjusted helmet, which would be more comfortable than a “rigid-mounted” CROM instrument. Third, an electronically displayed inclinometer would offer higher precision and legibility than the traditional pointer scale; moreover, parameter readings are both easy and rapid. However, the reliability and between-tester variability of our measuring apparatus require further study for validation.

The CSA of the deep cervical extensors, such as the multifidus, has found to be smaller in patients with CNNP [48]. Moreover, variable findings have been observed in the CSA of neck extensor muscles in patients with neck pain, including fatty infiltration in the deep cervical extensors [49], a increased CSA of the semispinalis capitis and splenius capitis [18], a decreased CSA of the semispinalis capitis muscle [19], and no change in CSA of the longissimus capitis [50]. In the present trial, we measured the CSAs of multifidus, semispinalis cervicis, semispinalis capitis, and splenius capitis on T1 magnetic resonance images. Extensive fatty infiltration of muscle tissue was not observed at each follow-up, possibly attributed to the relatively young age of the enrolled participants.

In the observation group, a noteworthy increase in the CSAs of cervical extensors was observed at each follow-up, encompassing both superficial and deep layers. The deep cervical extensors, including semispinalis and cervicis multifidus, primarily attach to C2 [51]. In an intramuscular electromyography study by Schomacher et al. [39], they observed no increase in activity in semispinalis cervicis relative to the splenius capitis when the subject pushed into extension against resistance applied to the occiput in a neutral occipitocervical position. However, when manual static resistance was applied to the posterior C2, the semispinalis cervicis was selectively activated relative to the splenius capitis. This activation was not observed when the resistance was applied at C5. In the present study, for the convenience of at-home exercise, we instructed participants to apply resistance slightly below the occiput, near the C3-C4 spinous process. Our findings demonstrated that the modified isometric exercise effectively activated the deep cervical extensors.

In the control group, we observed a slight increase in the CSAs of extensors, with increments of 3.26, 4.23, and 4.57 mm^2^ at 3-, 6-, and 12-month follow-up, respectively. However, the changes in neck muscle CSAs were marginal, particularly between the 6-month and 12-month follow-ups, indicating that stretching exercises alone may not be effective in significant muscle mass gain. These findings are consistent with the results from Häkkinen et al.’s trial [31], where neck stretching did not prove effective in improving muscle strength. We infer that the observed increase in muscle size among participants in the control group may be attributed to the alleviation of pain and improvement in disability, leading to enhanced daily neck movements and subsequent strengthening of neck muscles.

There is a widespread consensus that the ideal state for the cervical spine is characterized by a lordotic curvature [52]. Although the association between cervical lordosis loss and neck pain is a topic of debate, it has been reported in several studies [53, 54]. Clinical factors, such as muscular spasms, congenital defects, and cervical muscle weakness, are correlated with the loss of the cervical lordotic curve [55, 56]. In patients with CNNP, the neck extensor is notably more weakened than the neck flexor [55]. Moreover, the isometric exercise of the neck extensor could potentially restore the lordotic angle of the cervical spine in individuals with neck pain and cervical lordosis loss [35]. However, the underlying rationale for how specific muscle strengthening might contribute to the restoration of cervical lordosis remains unexplored.

In this trial, 28% of participants in the observation group restored cervical lordosis, compared with 9% in the control group at the 12-month follow-up. The between-group difference was marginal and non-significant, despite a significant increase in the CSAs of cervical extensors in the observation group. These findings align with the results of Yoon et al.’s trial [52], where they observed no correlation between the cervical lordosis angle and individual neck muscle CSAs; however, there was a positive correlation with the ratio of flexor to extensor muscles. These results suggest that restoring the balance between flexor and extensor muscles may be crucial for maintaining the physiological cervical lordotic curvature. The restoration of cervical lordosis in participants from the control group may be attributed to the improvement of neck pain and function. Several clinical trials using no-load usual exercises (such as extension traction) found increased cervical lordosis in participants with CNNP after pain relief and functional improvement [57].

While randomized controlled trials provide a high level of clinical evidence and are considered the gold standard for evaluating clinical effects, the present study has certain limitations that should be acknowledged. The relatively high dropout rate at the last follow-up can be attributed to the design of the long-term exercise program. Although mixed-effects models allow for partial data loss, we underestimated the dropout rate in such a long-term exercise protocol, highlighting the need for a larger sample size in future research. Owing to the limited numbers of experienced practitioners and long duration of training protocol, two researchers who were involved in the randomization process participated in the intervention, which potentially resulted in performance bias. Despite pharmacologic therapies often being included in conservative care for patients with CNNP, and some trials focusing on neck exercise also including drug use [30, 35], the short-term use of medication in this trial may potentially influence the effectiveness of the exercise. For the easy and rapid measurement of CROM, a specifically-made apparatus was utilized in this trial. While we hypothesized that the precision of measurement is high with a digital-displayed device, the reliability and between-tester variability require future study for confirmation. In the present trial, measurements of muscle strength and the extent of muscle activation were not included. Consequently, the underlying mechanism of how increased muscle CSAs are transformed into better cervical curvature remains unknown.

## Conclusion

We herein implemented a long-term specific training program for the cervical extensors in young adults with CNNP. Our findings indicate that specific cervical extensor exercises are superior to usual stretching exercises in alleviating neck disability and pain and enhancing CROM among young adults. Furthermore, specific training of cervical extensors in young adults significantly increases muscle CSAs, potentially contributing to the restoration of cervical lordosis.

## Data Availability

The data analyzed during the current study are available from the corresponding author upon reasonable request.

## Abbreviations

CNNP: Chronic non-specific neck pain
NDI: Neck disability index
VAS: Visual analog scale
CROM: Cervical range of motion
CSAs: Cross-sectional areas
